# Identifying and Evaluating Mobile and Web Apps for Patients to Manage Hidradenitis Suppurativa: Systematic Search in App Stores and Content Analysis

**DOI:** 10.2196/69030

**Published:** 2025-08-29

**Authors:** Caroline Glatzel, Tassilo Dege, Bernadette Glatzel, Matthias Goebeler, Dagmar Presser, Astrid Schmieder

**Affiliations:** 1 Department of Dermatology, Venereology and Allergology, University Hospital Würzburg, Josef-Schneider-Strasse 2, Würzburg, 97080, Germany, 49 931 201 26369, 49 931 201 26700

**Keywords:** hidradenitis suppurativa, acne inversa, mobile health apps, MHA, mobile apps, Mobile Application Rating Scale, MARS, uMARS, German mHealth App Usability Questionnaire, G-MAUQ, Mobile Device Proficiency Questionnaire, MDPQ-16, Affinity for Technology Interaction Scale, ATI Scale, skin

## Abstract

**Background:**

Hidradenitis suppurativa (HS) is a chronic inflammatory skin disease characterized by painful nodules, abscesses, and fistulas in intertriginous sites. It significantly impacts patients’ quality of life. Early diagnosis and timely treatment are essential for disease control. Recurrent flares, suboptimal therapies, and prolonged misdiagnosis place a significant burden on both patients and health care systems.

**Objective:**

We aimed to identify mobile health apps (MHAs) for patients with HS and evaluate their quality through assessments by both patients and physicians.

**Methods:**

Two reviewers searched for mobile and web apps for HS, including those only available in German or English. Apps with advertising or non–patient-centered content and apps related to trials or conferences were excluded. Two apps met the criteria and were evaluated by 20 physicians and 27 patients using the Mobile App Rating Scale (MARS), user version of the MARS (uMARS), German Mobile App Usability Questionnaire, and technology affinity tools (Affinity for Technology Interaction Scale and Mobile Device Proficiency Questionnaire).

**Results:**

We identified 2 apps for managing HS that met the inclusion criteria—the HSR-Patients app and the EHSF-Hidradenitis Suppurativa app—from an initial pool of 29 proposed apps that included many nonmedical, non–HS-specific, and non–patient-centered apps. Patients rated the quality of the HSR-Patients app significantly higher than physicians (MARS: mean 3.01, SD 0.60 vs. uMARS: mean 3.53, SD 0.69; *P*=.009). In contrast, ratings for the EHSF-Hidradenitis Suppurativa app did not differ significantly (physicians: mean 2.81, SD 0.55; patients: mean 2.72, SD 0.79; *P*=.69). Usability, assessed with the German Mobile App Usability Questionnaire, showed no significant difference between physicians and patients for either app. For the HSR-Patients app, physicians and patients rated usability at 4.37 (SD 0.86) and 4.72 (SD 1.21; *P*=.27), respectively. For the EHSF-Hidradenitis Suppurativa app, physicians and patients rated usability at 3.88 (SD 0.77) and 3.38 (SD 1.35; *P*=.11), respectively. Patients showed a significantly higher general affinity for technology than physicians, as measured by the Affinity for Technology Interaction Scale (physicians: mean 3.62, SD 0.61; patients: mean 4.38, SD 1.30; *P*=.01). However, there was no significant difference in affinity for technology specifically when using mobile devices, as assessed by the Mobile Device Proficiency Questionnaire (physicians: mean 4.83, SD 0.25; patients: mean 4.69, SD 0.72; *P*=.41).

**Conclusion**s**:**

This evaluation highlights the limited availability of high-quality, HS management–specific MHAs and underscores the need for more targeted digital tools. Differences in evaluations between patients and physicians were evident, with patients focusing on usability and practical guidance, while physicians prioritized content and usability. Neither the HSR-Patients app or the EHSF-Hidradenitis Suppurativa app demonstrated sufficient potential for long-term use, indicating the need for participatory development that includes all stakeholders.

## Introduction

### Background

Hidradenitis suppurativa (HS; also known as acne inversa) is a chronic, recurrent skin disease characterized by painful inflammatory nodules, abscesses, and tunnels in areas such as the axilla, groin, and genitalia. HS leads to a significant physical and psychological burden for those affected [1]. The global prevalence of HS is reported to be between 0.4% and 1%, with many cases remaining unreported [2,3]. Early and stage-appropriate treatment is essential to prevent irreversible tissue damage [4]. Chronic skin inflammation is frequently associated with relevant comorbidity including metabolic syndrome, chronic inflammatory bowel disease, axial spondyloarthropathies, and depression, which require interdisciplinary management. There is also a clear association with active smoking and obesity [3].

HS often manifests after puberty, significantly impacting patients’ lives due to chronic pain, limited mobility, persistent secretions, and unpleasant odors [5].

The diagnosis of HS is often considerably delayed, with a mean time to diagnosis of approximately 10 years from symptom onset reported in the literature [6]. While delays are sometimes attributed to patients, due to factors such as embarrassment or long waiting times for specialist consultations, it is important to recognize that diagnostic challenges in clinical practice may also play a role. In primary care, early HS lesions can resemble common skin infections and may be misinterpreted as such [3]. Smaller fistulas and abscesses are not always identified as manifestations of HS and may be diagnosed and treated as isolated dermatological findings [7,8]. Thus, patients often undergo multiple surgeries for recurrent abscesses, which greatly reduces their quality of life [9].

In the search for answers and support, many patients turn to the internet. Once a diagnosis has been made, HS-specific mobile health apps (MHAs) can serve as valuable tools for both patients and physicians. These apps may help improve medical care by supporting patients’ education, explaining complex aspects of HS, enabling symptom tracking, and facilitating communication. This can empower patients to better manage their condition and help physicians save time during consultations. To fully leverage this potential, the integration of MHAs into routine care for HS should be further explored and promoted.

MHAs or digital health apps (DiGAs) already offer a promising opportunity to support self-management [10] of diseases and ideally have a positive impact on care. Since 2019, specially certified, patient-centered MHAs can be prescribed in Germany as DiGAs, which are covered by public health insurances. These DiGAs support disease self-management, improve care coordination, facilitate access to health care, enhance health literacy, and provide guideline-based recommendations. They also allow users to track disease progression (eg, through photo documentation and questionnaires), monitor therapy progress, and access helpful resources during acute episodes, ultimately improving disease control and quality of life. Germany, with its large insured population, is an attractive market for health care digitalization. However, there are currently no empirically validated DiGAs specifically developed for patients with HS, highlighting the need for a thorough evaluation of available MHAs. Given the large number of apps on the market, critical evaluation is essential [11]. The quality and effectiveness of apps can vary widely, and few offer the features that patients and clinicians actually need. Collecting user experiences and feedback is therefore vital for identifying the best digital tools for patients with HS [12,13].

### Aim of This Study

The aims of this study were to analyze the quality and usability of currently available apps, to gain insight into users’ technology affinity and requirements, and to draw conclusions for the development of future HS apps as DiGAs. We sought to answer these questions using validated questionnaires, including the Mobile Application Rating Scale (MARS), German mHealth App Usability Questionnaire (G-MAUQ), Affinity for Technology Interaction (ATI) Scale, Mobile Device Proficiency Questionnaire (MDPQ-16), and an open-ended questionnaire developed specifically for this study.

We hypothesize that there are currently no high-quality HS apps available in app stores, but the development of such apps will be essential in the future—not least due to the high technology affinity of patients with HS. We believe that patients with HS, who are typically young and tech-savvy, would greatly benefit from a well-designed, customized app that tracks their therapy, documents involved anatomical locations for better treatment control, provides support during acute stress situations, and strengthens the connection with their health care providers.

## Methods

### Ethical Considerations

This analysis was conducted in accordance with the Declaration of Helsinki and ethics approval was obtained from the local ethics committee of the University of Würzburg (2023091901). All participants provided written informed consent. Privacy and confidentiality were maintained in accordance with applicable data protection regulations.All personal data were anonymized and securely stored. No compensation was provided for participation.

### App Selection

Two reviewers systematically and independently searched the Apple App Store, the Google Play Store, and the internet for mobile apps designed for patients with HS from November to December 2023. The following search terms were used: *hidradenitis suppurativa*, *acne inversa*, *abscesses*, *furuncles*, *fistulas*, *skin condition*, and *wound*. The inclusion criteria required the apps to be available in one of the aforementioned app stores and in either German or English. The exclusion criteria included apps that contained advertising; were not patient oriented, which means they were intended for health care professionals rather than patients; or were related to clinical trials or conferences. In total, 29 apps were identified, of which 27 apps were excluded for the following reasons: not patient centered (n=2; not focusing on patients’ needs), not available in English or German (n=1), not specific to HS (n=24), and containing advertisements (n=4). After applying the inclusion and exclusion criteria, 2 apps met the requirements: the HSR-Patients app and the EHSF-Hidradenitis Suppurativa app. The HSR-Patients app was available in both the Apple App Store and the Google Play Store and in the German language. The EHSF-Hidradenitis Suppurativa app was only available in the Apple App Store and only in the English language. No mobile apps for patients with HS were found on the internet. Patients and physicians tested the app version corresponding to the operating system available on their personal smartphones (iOS or Android). If they did not own an iOS device, the EHSF-Hidradenitis Suppurativa app, available only for iOS, was tested using a study-provided iPhone to ensure both apps could be evaluated by all participants.

Information about the identified apps was systematically collected, including the app name, platform availability, target audience, language, app store rating, number of reviews, chargeability, presence of advertising, and developer details.

### Evaluation of App Quality

The MARS is one of the most commonly used scores to evaluate the quality of mobile apps [14]. The MARS score is calculated based on 19 questions in 4 different categories (engagement, functionality, aesthetics, and information). Each item is rated on a 5-point Likert scale. Higher scores indicate better quality. The overall MARS score is calculated as the mean of the 4 objective subscales. The German translation of the MARS, the validated MARS-G, was used in this study [15]. For readability, the term “MARS” is used throughout the manuscript to refer to the validated German version (MARS-G) applied in this study.

The uMARS is a simplified end-user version of the MARS that reliably measures the quality of MHAs [16]. The German translation of the uMARS used in this study has not yet been formally published; however, its items are identical to those in the validated MARS-G.

### Evaluation of App Usability

The G-MAUQ [17]—the German, validated version of the MAUQ [11]—is a tool to determine the usability of an MHA. It measures the ease of use, the user’s satisfaction with the design of the app (interface and satisfaction), and the usefulness of an MHA for the user. The questionnaire has 18 questions that are to be answered on a 7-point Likert scale. Higher scores indicate better usability. Unlike the MARS score, it was developed specifically for evaluating medical apps [18].

The 2 apps suitable for patients with HS were evaluated by a total of 20 physicians using the MARS-G for quality and the G-MAUQ for usability [19]. The same apps were rated simultaneously by 27 patients with HS. They used the uMARS and evaluated the usability of the apps using the G-MAUQ. Before evaluating the apps using the questionnaires, the testers were asked to test the apps for at least 10 minutes each.

### Evaluation of Technical Affinity

In addition, physicians’ and patients’ affinity for technology was assessed using the ATI Scale and the MDPQ-16. The ATI Scale, a 9-item scale, is a widely used, well-established tool for quantifying people’s general affinity for technology [20]. For the ATI Scale, higher scores indicate a greater affinity for technology. In addition, we used the MDPQ-16, a short version of the MDPQ, and also asked about affinity for technology, specifically when using mobile devices [21]. For the MDPQ-16, higher scores indicate greater proficiency in using mobile devices.

### Evaluation of Patients’ Need for a Patients’ App

To explore the potential role of digital support tools in the management of HS, both patients and physicians were asked to complete a questionnaire specifically developed for this study. It included open-ended questions addressing current experiences, unmet needs, and expectations regarding MHAs tailored to HS. The aim was to assess overall interest in digital support and to identify key features that could enhance disease monitoring, communication, and self-management.

### Comparative Analysis of Patients’ and Physicians’ Data

Following the evaluation of MARS, uMARS, G-MAUQ, ATI Scale, and MDPQ-16, we performed a comparative analysis including physicians and patients. Calculations were performed using SPSS (version 23; IBM Deutschland GmbH). After testing for normal distribution, either the Mann-Whitney *U* or 2-tailed *t* test was used, with *P* values <.05 considered statistically significant.

## Results

### App Selection

**Figure 1.**
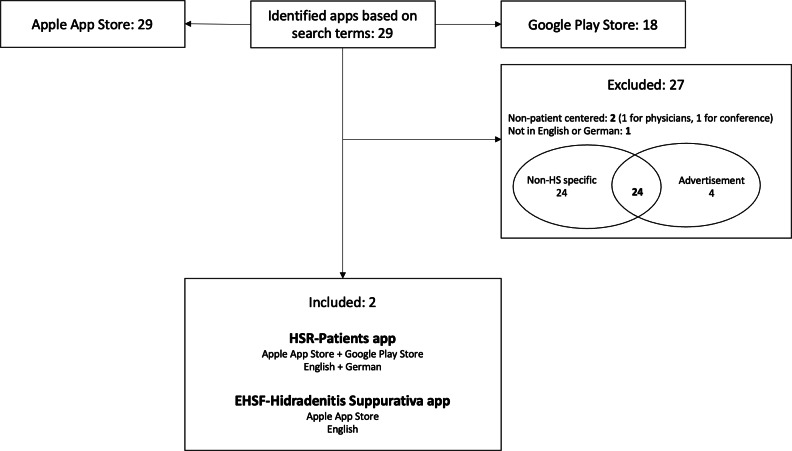
Flowchart illustrating the process of app selection. HS: hidradenitis suppurativa.

From a total of 29 proposed apps, which included many nonmedical, non–HS-specific, and non–patient-centered apps, the 2 currently available patient-centered HS apps were identified: the HSR-Patients app and the EHSF-Hidradenitis Suppurativa app (Figure 1). The HSR-Patients app was developed by swiss4ward Europe S.L. with support from the Swiss Hidradenitis Suppurativa Foundation. It is available in English, French, German, and Spanish and serves as an information resource for patients with HS. Last updated in 2020, it offers various sections where patients can access information about HS, including “What is Hidradenitis Suppurativa,” “Definition,” “Lifestyle Adjustments,” “Dietary Changes for HS,” “Important Points for Effective Wound Management,” and “Intimate Lesions.”

The EHSF-Hidradenitis Suppurativa app is a Conformité Européenne–certified, English-language app developed in 2018 by the European Hidradenitis Suppurativa Foundation e.V. in collaboration with everywhereIM BV. It supports HS classification based on the “refined Hurley classification” [22] and presents treatment options. In the “Classification” tab, patients can indicate the number of body parts affected by HS, the number of abscesses or inflammatory nodules, and the presence of tunnels. Based on these data, the patient is shown the Hurley stage and appropriate therapy suggestions. The patient can then export a summary of this information as a PDF document. There are 2 references under the “Literature” tab. Only one tab can be opened at a time. The patient can access information on “Refined Hurley Staging,” while the other tab IHS-4 cannot be opened at the same time.

### Raters’ Characteristics

A total of 20 physicians and 27 patients affected by HS for at least 1 year evaluated the 2 apps. The average age of the physicians was 31.5 (SD 3.12) years, while the average age of the patients was 41.8 (SD 9.5) years. There were 7 men and 13 women among the physicians and 15 men and 12 women among the patients with HS.

### Evaluation of App Quality

A total of 2 apps, the HSR-Patients and the EHSF-Hidradenitis Suppurativa apps, met the inclusion criteria and were included in this analysis. The results of the MARS and uMARS scores for the respective apps are shown in Tables1  2.

The quality of the HSR-Patients app was assessed by physicians using the MARS and by patients using the uMARS. Higher scores indicate a better result. Physicians rated the app’s quality significantly lower than patients, with a mean score of 3.01 (SD 0.60) out of a possible 5 points, compared to the patients’ average rating of 3.53 (SD 0.69), indicating a statistically significant difference (*P*=.009).

In all uMARS subcategories—engagement, functionality, aesthetics, information, and subjective quality—patients consistently rated the HSR-Patients app higher than the EHSF-Hidradenitis Suppurativa app. Notably, there were significant rating differences between physicians and patients for the HSR-Patients app in the aesthetics (*P*=.008) and information (*P*=.04) categories. In both categories, patients rated the app significantly higher than physicians.

**Table 1.** MARS score of the HSR-Patients app and EHSF-Hidradenitis Suppurativa app assessed by 20 physicians.

		HSR-Patients app	EHSF-Hidradenitis Suppurativa app
MARS score^b^, mean (SD)		3.01 (0.60)	2.81 (0.55)
	A: engagement score	2.18 (0.54)	2.20 (0.66)
	B: functionality score	4.21 (0.58)	3.68 (0.77)
	C: aesthetics score	2.97 (0.72)	2.65 (0.63)
	D: information score	2.99 (0.67)	3.05 (0.77)
	PT: psychotherapy score	2.85 (0.51)	2.82 (0.76)
	E: subjective quality	2.13 (0.55)	1.98 (0.72)

a MARS: Mobile Application Rating Scale.

b Maximum MARS score: 5 points.

**Table 2.** uMARS^a^ score of the HSR-Patients app and EHSF-Hidradenitis Suppurativa app assessed by 27 patients with hidradenitis suppurativa.

		HSR-Patients app	EHSF-Hidradenitis Suppurativa app
uMARS score^b^, mean (SD)		3.53 (0.69)	2.72 (0.79)
	A: engagement score	2.50 (0.81)	2.16 (0.95)
	B: functionality score	4.37 (0.59)	3.26 (1.02)
	C: aesthetics score	3.58 (0.76)	2.63 (0.83)
	D: information score	3.35 (0.79)	2.84 (1.02)
	E: subjective quality	2.31 (0.88)	1.77 (0.83)

a uMARS: user version of the Mobile Application Rating Scale.

b Maximum uMARS score: 5 points.

### Evaluation of Usability

Usability of the HSR-Patients app was rated similarly by physicians and patients. Higher scores are correlated with better results. The G-MAUQ for the usability of the HSR-Patients app was on average 4.37 (SD 0.86) out of 7 points for physicians and 4.72 (SD 1.21) out of 7 points for patients. There was no significant difference between physicians and patients (*P*=.27; Tables3  4).

The EHSF-Hidradenitis Suppurativa app received lower overall usability ratings than the HSR-Patients app. Although patients rated the usability of the English-language EHSF-Hidradenitis Suppurativa app to be lower than physicians, this difference was not statistically significant (*P*=.11). Physicians gave the EHSF-Hidradenitis Suppurativa app a mean usability score of 3.88 (SD 0.77), while patients rated it at 3.38 (SD 1.35). However, a significant difference was found between physicians and patients in the ease of use category. Patients rated the app’s ease of use significantly lower, with a mean score of 4.10 (SD 1.74), than the physicians’ higher rating of 4.91 (SD=0.75), reflecting a statistically significant difference (*P*=.04).

**Table 3.** Evaluation of G-MAUQ^a^ and G-MAUQ subscales by physicians.

		HSR-Patients app	EHSF-Hidradenitis Suppurativa app
G-MAUQ score^b^, mean (SD)		4.37 (0.86)	3.88 (0.77)
	Ease of use	5.77 (0.69)	4.91 (0.75)
	Interface and satisfaction	3.82 (1.19)	3.59 (1.20)
	Usefulness	3.71 (1.09)	3.31 (1.00)

a G-MAUQ: German mHealth App Usability Questionnaire.

b Maximum G-MAUQ score: 7 points.

**Table 4.** Evaluation of G-MAUQ^a^ and G-MAUQ subscales by patients.

		HSR-Patients app	EHSF-Hidradenitis Suppurativa app
G-MAUQ score^b^, mean (SD)		4.72 (1.21)	3.38 (1.35)
	Ease of use	6.07 (1.00)	4.10 (1.74)
	Interface and satisfaction	4.65 (1.59)	3.40 (1.55)
	Usefulness	3.66 (1.60)	2.84 (1.54)

a G-MAUQ: German mHealth App Usability Questionnaire.

b Maximum G-MAUQ score: 7 points.

### Evaluation of Technical Affinity

The ATI Scale score and the MDPQ-16 score, which assess the user’s affinity for technology, were collected from both patients and physicians. The higher the ATI Scale and MDPQ-16 scores, the higher the affinity for technology.

Physicians rated their overall affinity for technology with an ATI Scale score of 3.62 (SD 0.61) out of 6 points and with an MDPQ-16 score of 4.83 (SD 0.25) out of 5 points. Patients rated their affinity for technology with an ATI Scale score of 4.38 (SD 1.30) out of 6 points and with an MDPQ-16 score of 4.69 (SD 0.72) out of 5 points.

A statistically significant difference was found between physicians and patients in their overall affinity for technology, as measured by the ATI Scale (physicians: mean 3.62, SD 0.61; patients: mean 4.38, SD 1.30; *P*=.01), with patients rating themselves as more technology oriented. However, no significant difference was observed between physicians and patients in their affinity for technology when using mobile devices, as measured by the MDPQ-16 (physicians: mean 4.83, SD 0.25; patients: mean 4.69, SD 0.72; *P*=.41).

### Patients’ Expectations and Comments

Most patients with HS expressed interest in using an app to support the management of their condition. Specifically, 85% (23/27) of patients indicated that they could envision using such an app if it provided a “value-added, targeted service.” If these criteria were met, 93% (25/27) of patients stated they would be willing to use the app regularly as a constant companion.

The most frequently mentioned features included symptom diary (21/27, 78%) for documenting therapy outcomes, identifying trigger factors, and monitoring quality of life and photo documentation (19/27, 70%) for tracking the condition’s progression.

All patients expected the app to provide “useful information for daily life,” with a particular emphasis on it being “regularly updated” and delivering “the latest insights on HS” in an easily understandable format. During acute flare-ups, patients wanted the app to offer “personalized tips and instructions for symptom relief” (such as pain management) in “clear language.”

The “constant availability” of the app was highly valued, as it would allow users to access information and support at any time, offering guidance on therapy and disease prevention, complementing health care provider visits. In addition, the app should facilitate “easy, straightforward contact with a physician” during acute situations.

About half of the patients (14/27, 52%) expressed interest in receiving an introduction to the app and assistance with using the technology. Furthermore, 41% (11/27) of patients desired support from a digital companion to guide them through the app’s features.

## Discussion

### Principal Findings

MHAs are increasingly being used as digital therapeutics in the medical field. As their use grows, so does the need for a robust and standardized evaluation of these apps [11].

A systematic inventory and assessment of currently available medical apps, using established and validated evaluation tools, is essential for obtaining feedback from both users (ie, patients) and prescribers (ie, health care professionals) [23]. This feedback is crucial for the continuous improvement of MHAs. The better an MHA aligns with user needs, the higher the likelihood of sustained engagement and long-term use [24].

We present the first systematic analysis of MHAs available for patients with HS in app stores and on the web. For all instruments used (eg, MARS, MARS-G, uMARS, ATI Scale, and G-MAUQ), higher scores consistently indicate better results.

The range of MHAs for managing HS is still very limited, with only 2 out of the 29 proposed apps meeting the inclusion and exclusion criteria. This reflects a broader gap in the availability of HS-specific digital tools and highlights the need for further development and evaluation of mobile health solutions tailored to the needs of this patient population.

The quality of the included apps was assessed by physicians and patients using the validated and widely used MARS score [16,18], and the overall score (mean MARS and uMARS score) ranged from a minimum of 2.72 to a maximum of 3.53 out of 5 possible points.

At first glance, the HSR-Patients app appeared to be the superior option of the two. Both physicians and patients rated it higher than the EHSF-Hidradenitis Suppurativa app across nearly all categories. However, physicians rated the app significantly lower than patients (*P*=.009).

Patients and physicians particularly appreciated the ease of use (ie, functionality) of the HSR-Patients app. Patients also rated the app highly for its appealing design (ie, aesthetics) and content (ie, information). However, physicians rated the information content (*P*=.04) and aesthetics (*P*=.008) of the HSR-Patients app significantly lower than patients.

One possible explanation for this discrepancy could be that some practical tips for daily life in the app did not align with current medical practice, a factor more apparent to physicians with medical knowledge. Patients who are lacking this expertise are less able to verify the accuracy of the content.

Previous literature has shown that patients tend to trust the content of medical apps more than physicians [25]. As a result, the app’s design may have been of greater importance to patients than to health care providers.

The HSR-Patients app’s attractiveness (engagement and subjective quality)—that is, the motivation to use the app repeatedly over time or to recommend it to others—was rated as low by both physicians and patients. Once the initial content had been read, there was no additional benefit in the second review. Only looking up individual facts could motivate the patient to use the app again. However, long-term use is important to achieve good results in maintaining the patient engaged with the MHA [25].

In contrast, the EHSF-Hidradenitis Suppurativa app offered slightly more interactivity. As the severity of HS progressed, users could adjust therapy options by entering them into the app’s interactive scoring system.

Regular monitoring and, when necessary, adjusting therapy based on disease severity is strongly recommended in the S2k guideline to ensure treatment adherence [5].

This feature in the EHSF-Hidradenitis Suppurativa app may explain the higher ratings from physicians in the “information” subcategory.

Unlike patients, physicians were able to quickly categorize and apply the information provided by the EHSF-Hidradenitis Suppurativa app during the 10-minute testing phase. For physicians, the app’s design was of secondary importance.

For patients, an appealing design, good functionality, and practical information and tips for everyday use seem to be the fundamental requirements for the successful integration of an MHA into routine medical practice.

In addition, documenting the disease course in the app could help physicians make more informed decisions about systemic therapy. By tracking the progression of the disease over time, physicians might categorize patients as moderate to severe HS, even if they would not qualify based on a single scheduled assessment (eg, by IHS4).

The results from the G-MAUQ (usability) supported this finding. Overall, both physicians and patients rated the HSR-Patients app as more user-friendly (mean G-MAUQ). The design (ie, interface), patient satisfaction with the app’s functions, and the perceived usefulness of the app were rated between 2.84 and 3.59 out of a possible 7 points by both groups. Both physicians and patients gave the highest ratings for ease of use. However, patient satisfaction was lower (*P*=.04), likely due to the challenge of using an app in a foreign language and the novelty of classifying HS, which presented a greater barrier for patients than for physicians.

Overall, patients with HS rated themselves as more tech-savvy than physicians, according to the ATI Scale, a broader technology familiarity questionnaire (*P*=.01).

Physicians and patients rated themselves as having the same affinity for using MHAs. This suggests that a well-designed MHA would certainly be well received by both patients and health care providers.

A comparison of our results with MHA assessments in other chronic dermatological conditions has revealed interesting findings.

For example, in a recent study on wound care mobile apps, both patients and physicians rated the quality of an MHA as moderate, with a mean MARS score of 3.88 and a mean uMARS score of 3.89, suggesting relatively aligned expectations. In contrast, in our HS study, patient ratings of the HSR app (uMARS: mean 3.53, SD 0.69) were significantly higher than those of physicians (MARS: mean 3.01, SD 0.60), indicating a greater divergence in perceived value and user priorities [23].

A more detailed investigation of the subcategory ratings reveals that the WUND App was rated less favorably by physicians in the “information” subcategory and by patients in the “functionality” subcategory, which is consistent with our findings that physicians tend to prioritize content quality while patients place greater emphasis on usability and practical relevance [23].

In the wound care study, patients rated their technical affinity (ATI Scale) slightly lower than physicians, although the difference was not statistically significant. In contrast, patients with HS in our cohort reported significantly higher ATI Scale scores than their physicians. This difference may reflect demographic factors, such as the younger average age of patients with HS, and highlights the need to tailor MHAs to the digital affinity of different patient groups.

In a systematic evaluation of MHAs for psoriasis, the app Psoriasis Helferin was rated significantly lower by patients (uMARS: mean 3.48) than by physicians (MARS: mean 4.18), with lower scores across all subcategories. The authors emphasized that this discrepancy underscores the importance of involving patients in both the development and evaluation of MHAs, as perceptions of quality and usefulness may differ considerably between end users and health care professionals [26].

The lack of co-design and user-centered development in most currently available MHAs contributes to inconsistencies and hinders the effective development and continuous improvement of MHAs. This is particularly evident in the HS apps we analyzed, which demonstrated limited potential for long-term usability and user acceptance. To address these shortcomings, future HS-specific app development should be guided by established regulatory frameworks that emphasize clinical relevance, user engagement, data protection, and technical interoperability. A structured co-design process—bringing together patients, dermatologists, app developers, and regulatory experts from the outset—could ensure the creation of digital tools that are both clinically effective and tailored to user needs and the requirements of modern health care systems.

### Limitations

The small number of included apps (n=2) represents a major limitation of this study. However, this does not primarily reflect a methodological weakness but rather reveals a substantial gap in the availability of HS-specific mobile health tools. This market deficiency underlines the need for increased attention to digital innovation in the care of patients with HS.

In most cases, the apps included in the study were tested by participants for 10 minutes each, allowing them to form an initial impression. However, it was not possible to draw conclusions regarding the long-term benefits of these apps. Further research is needed to assess the long-term effectiveness of MHAs for individuals with HS. Nevertheless, the 2 apps designed exclusively for patients with HS appear to be less suitable for this purpose.

### Conclusions

MHAs are viewed differently by patients and physicians. In this analysis, patients emphasized the importance of ease of use, an appealing interface, and practical information that helps manage their condition in everyday life. However, patients often face challenges in verifying the accuracy of the information provided by the app. Physicians, while also appreciating user-friendliness and a well-designed interface, prioritize the quality and accuracy of the content. Neither of the currently available apps appears to fully engage physicians or patients for long-term use. However, the EHSF-Hidradenitis Suppurativa app shows promise in supporting patients throughout their disease progression by personalizing treatment recommendations based on symptoms.

For an MHA to be more effective, it should combine an intuitive interface with interactive features that assess disease activity and allow for real-time treatment adjustments. In addition, the app should support a more individualized treatment approach, empowering physicians to offer tailored care rather than relying solely on scheduled check-ups. This would make the app not only a tool for disease management but also an extension of the patient-physician partnership.

Future development of HS-specific MHAs should be guided by participatory co-design approaches that actively involve patients and health care professionals to ensure usability, clinical relevance, and long-term acceptance.

## Abbreviations

ATI: Affinity for Technology Interaction
DiGA: digital health app
G-MAUQ: German mHealth App Usability Questionnaire
HS: hidradenitis suppurativa
MARS: Mobile Application Rating Scale
MARS-G: Mobile Application Rating Scale–German
MDPQ-16: Mobile Device Proficiency Questionnaire
MHA: mobile health app
uMARS: user version of the Mobile Application Rating Scale
